# Supplementary material for: An independently tunable dual control system for RNAi complementation in Trypanosoma brucei
Source: PLoS One. 2025 May 12;20(5):e0321334. doi: 10.1371/journal.pone.0321334 (PMC12068568; doi:10.1371/journal.pone.0321334)
Supplement: S1 Table — Underline letter; restriction enzyme site. (PDF) [file pone.0321334.s001.pdf]

Table S1: Primers used in this study.

Gene	Purpose	Primer Name	Primer Sequence (5' – 3')
Not applicable	pJ1271 site directed mutagenesis	UM113	CTT AAG CGC AGC GGG ATC GCT AAC GCG TAT GTG ATC CTT AGT CAA GTG G
		UM114	CCA CTT GAC TAA GGA TCA CAT ACG CGT TAG CGA TCC CGC TGC GCT TAA G
POLIB Tb927.11.4690	OE subcloning Dual Inducer System	UM117	CAC CAA AAA GTA AAA TTC ACA GAT GCG GCT AAA TAG CTG C
		UM118	AGC CAA CTA AAT GGG CAG CAT CAG GTT GAC TTC CC
	Northern blot	MK157	TAT GAG TCT AGA AAG ATG AGC GTG TCA ACG AGG
		MK159	TAT GAG ACG CGT GGT AAA CCG TGG CGC GAC GAG G
	Allelic tagging	MK351	TTG TGT <u>GGG CCC</u> GGC TAT CGA CAA GTC TCT CTC TC
		MK352	TGT TGT <u>CGG CCG</u> CAC CGT AAT TTC TAC ACT GTC
	Allelic Knockout	MK455	TAT AGA <u>CTC GAG</u> GTT GTT GTT TGC CCA CCG TTC G
		MK456	TAT AGA <u>AAG CTT</u> ATC ACT ATG CGG ACC ACC AG
		MK457	TAT ATA <u>ACT AGT</u> GAC ATT CCC AGG TGT TAA GTT G
		MK458	TAT ATA <u>TCT AGA</u> CAC TTC TGC CCT CGC CC
POLIC Tb927.7.3990	Northern blot	UM130	CAT TCA CAG GGG TTG AAG TCA TCG C
		UM131	ACG TTC CAC CCT ACT GTA CAC TAC G
POLID Tb927.11.3260	Northern blot	UM136	GCG TGA TTG CTT AGT AAG TTG GTG
		UM137	TAC GAA TCA GTG CCC AAG TGG AGC
VanR WP_010920250.1	Northern blot	UM125	GCC AGG TCA GCG TGT GAT GAT G
		UM126	TGC TCG TAC TCT GCT GGA AGA TCC

Underline letters; restriction enzyme site.
