# Supplementary material for: An independently tunable dual control system for RNAi complementation in Trypanosoma brucei
Source: PLoS One. 2025 May 12;20(5):e0321334. doi: 10.1371/journal.pone.0321334 (PMC12068568; doi:10.1371/journal.pone.0321334)
Supplement: S2 Table — Superscript refers to the inducer used for the cell line. Tet, tetracycline; Van, vanillic acid; VaT, vanillic acid and tetracycline. (PDF) [file pone.0321334.s002.pdf]

Table S2: Cell lines used in this study.

Name	Description	Drug Selection	Reference
Lister 427	Procyclic Wild Type	None	N/A
29-13	Lister 427<br>TetR/T7RNAP	G418, Hyg	Wirtz et al 1999
$IBRNAi^{Tet}$	Single Inducer<br>POLIB RNAi Clone 2C7	G418, Hyg, Phleo	Bruhn et al 2010
$IBOE^{Tet}$	Single Inducer<br>POLIB-PTP ectopic expression Clone P1D12	G418, Hyg, Puro	Delzell et al 2022
IBPTP/KO	Single Expressor POLIB-PTP Clone P2F11	G418, Puro	This study
$IBComp^{Tet}$	Single Inducer<br>POLIB RNAi + POLIB-PTP ectopic expression Clone P2G7	G418, Hyg, Phleo, Puro	This study
SMUMA	Lister 427 VanR/TetR/T7RNAP	Puro	This study
$IBRNAi^{Van}$	Dual Inducer<br>POLIB RNAi Clone P2G9	Puro, Bsd	This study
$IBRNAi^{Van}$:IBPTP	Dual Inducer<br>POLIB RNAi + POLIB-PTP POP	Puro, Bsd, G418	This study
$IBComp^{VaT}$	Dual Inducer<br>POLIB RNAi + POLIB-PTP ectopic expression Clone P2D11	Puro, Bsd, Phleo	This study

Superscript refers to the inducer used for the cell line. Tet, tetracycline; Van, vanillic acid; VaT, vanillic acid and tetracycline.
