# Supplementary figures and images for: An independently tunable dual control system for RNAi complementation in Trypanosoma brucei
Source: PLoS One. 2025 May 12;20(5):e0321334. doi: 10.1371/journal.pone.0321334 (PMC12068568; doi:10.1371/journal.pone.0321334)

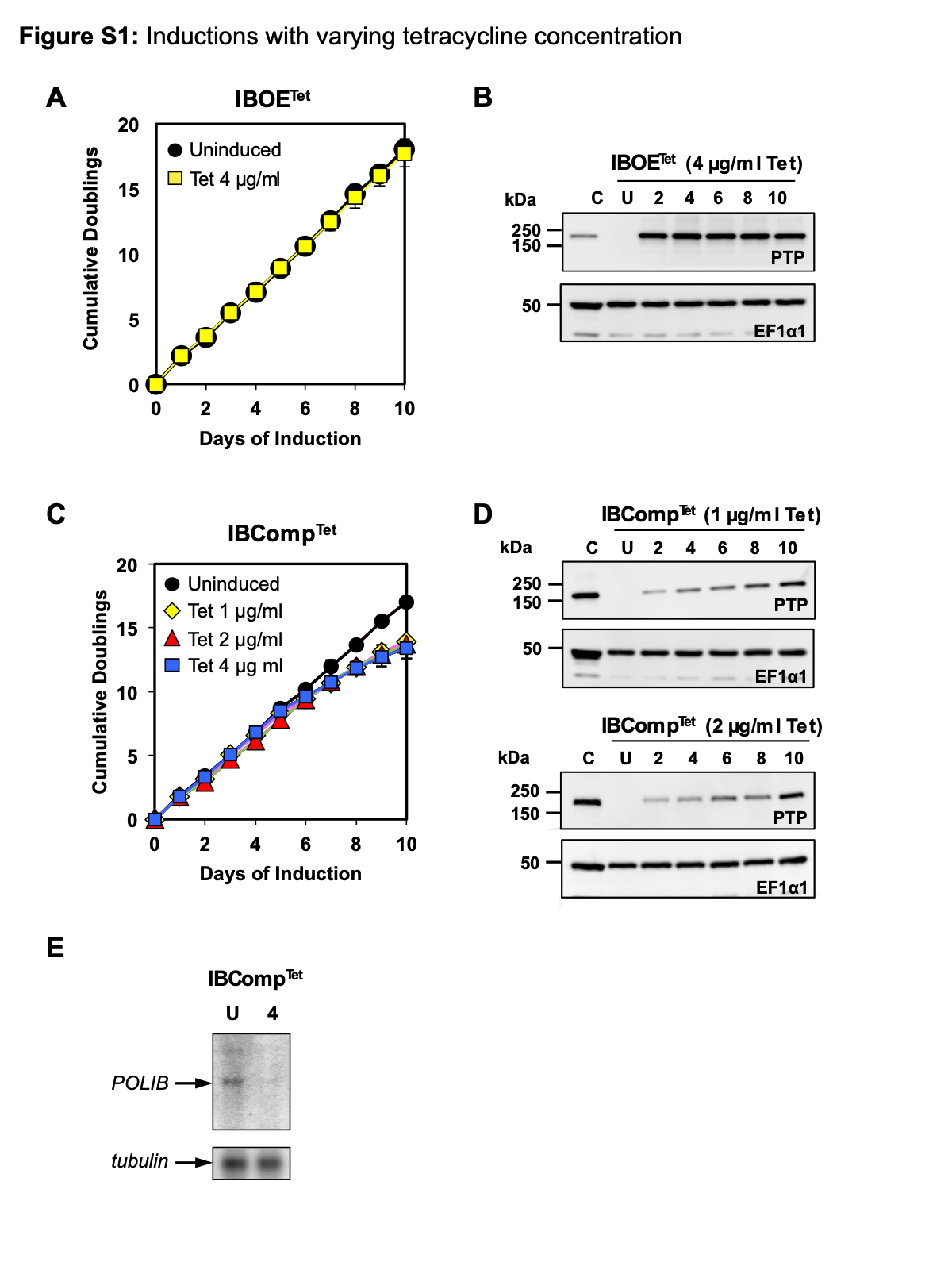

Supplement: S1 Fig — (A) Growth curve of IBOETet grown in the absence and presence of 4 µg/ml Tet. Error bars represent ± s.d. of the mean from three biological replicates. Some error bars are too small to be displayed. (B) Western blot detection of IBWT and EF1α1 during a 10 day induction. 2 x 10^6^ cell equivalents were loaded per lane. C, POLIB-PTP single allele tagged cell line. (C) Growth curve of IBCompTet grown in the absence and presence of 1, 2 and 4 µg/ml Tet. Error bars represent ±s.d. of the mean from three biological replicates. Some error bars are too small to be displayed. (D) Western blot detection of PTP tag and EF1α1. 2 x 10^6^ cell equivalents loaded per lane. C, POLIB-PTP single allele tagged cell line; 4 x 10^6^ cell equivalents. (E) Northern blot of total RNA from IBCompTet. U, uninduced; 4, induced with 4 µg/ml Tet for 48 hr. Following probing for TbPOLIB (4.2 kb), the same blot was stripped and reprobed for α-tubulin. (TIF) [file pone.0321334.s003.tif]

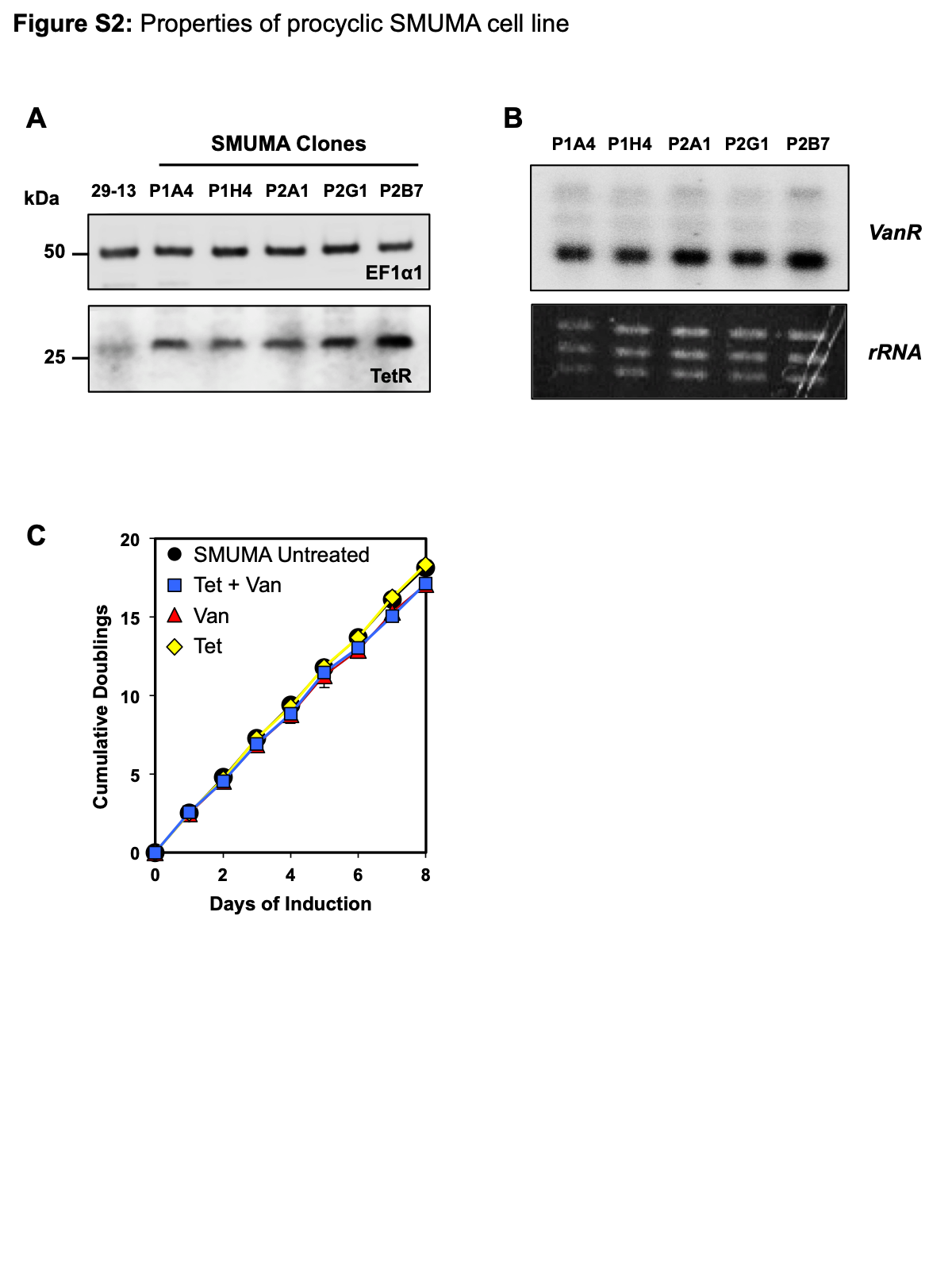

Supplement: S2 Fig — (A) Western blot detection of Tet repressor and EF1α1 in 29-13 and SMUMA clonal cell lines. 2 x 10^6^ cell equivalents loaded per lane. (B) Northern blot of total RNA from SMUMA clonal cell lines. Top, probing for the Van repressor; Bottom, EtBr-stained rRNAs as loading control. (C) Growth of SMUMA P2B7 clonal cell line in the absence and presence of 250 µM Van, 4 µg/ml Tet or a combination of both. Error bars represent ±s.d. of the mean from three biological replicates. Some error bars are too small to be displayed. (TIF) [file pone.0321334.s004.tif]

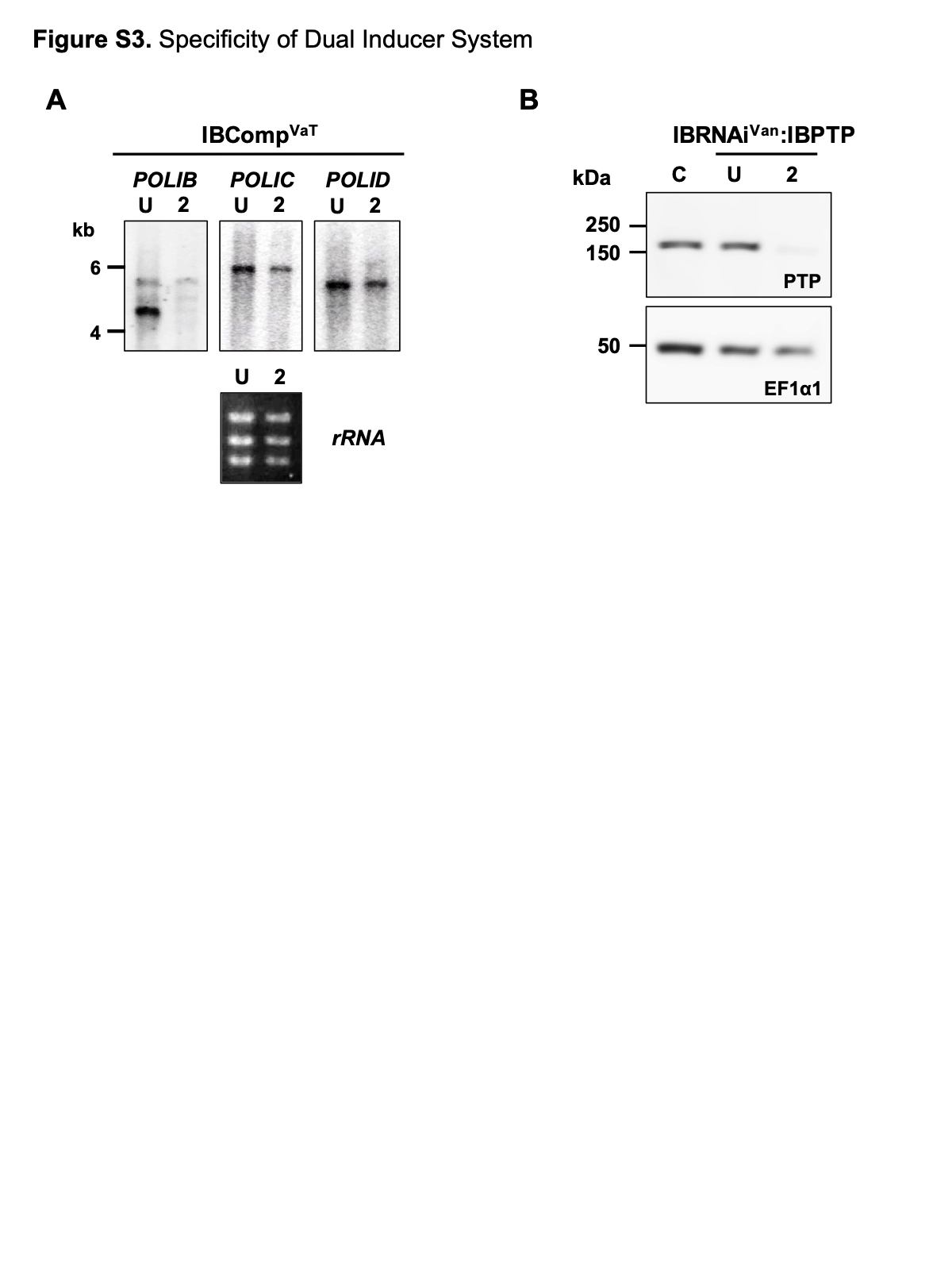

Supplement: S3 Fig — (A) Northern blot of total RNA from IBCompVaT clonal line P5D1. U, uninduced; 2, induced with 250 µ M Van for 48 hr. Following probing for TbPOLIB (4.2 kb), the same blot was stripped and reprobed for POLIC, POLID. EtBr-stained rRNAs as loading control. (B) Representative western blot detection of IBPTP and loading control EF1α1 from IBRNAiVan. C, single allele cell line endogenously expressing POLIB-PTP; U, uninduced; 2, induced with 250 µM Van for 48 hr. 5 x 10^6^ cell equivalents were loaded per lane. (TIF) [file pone.0321334.s005.tif]

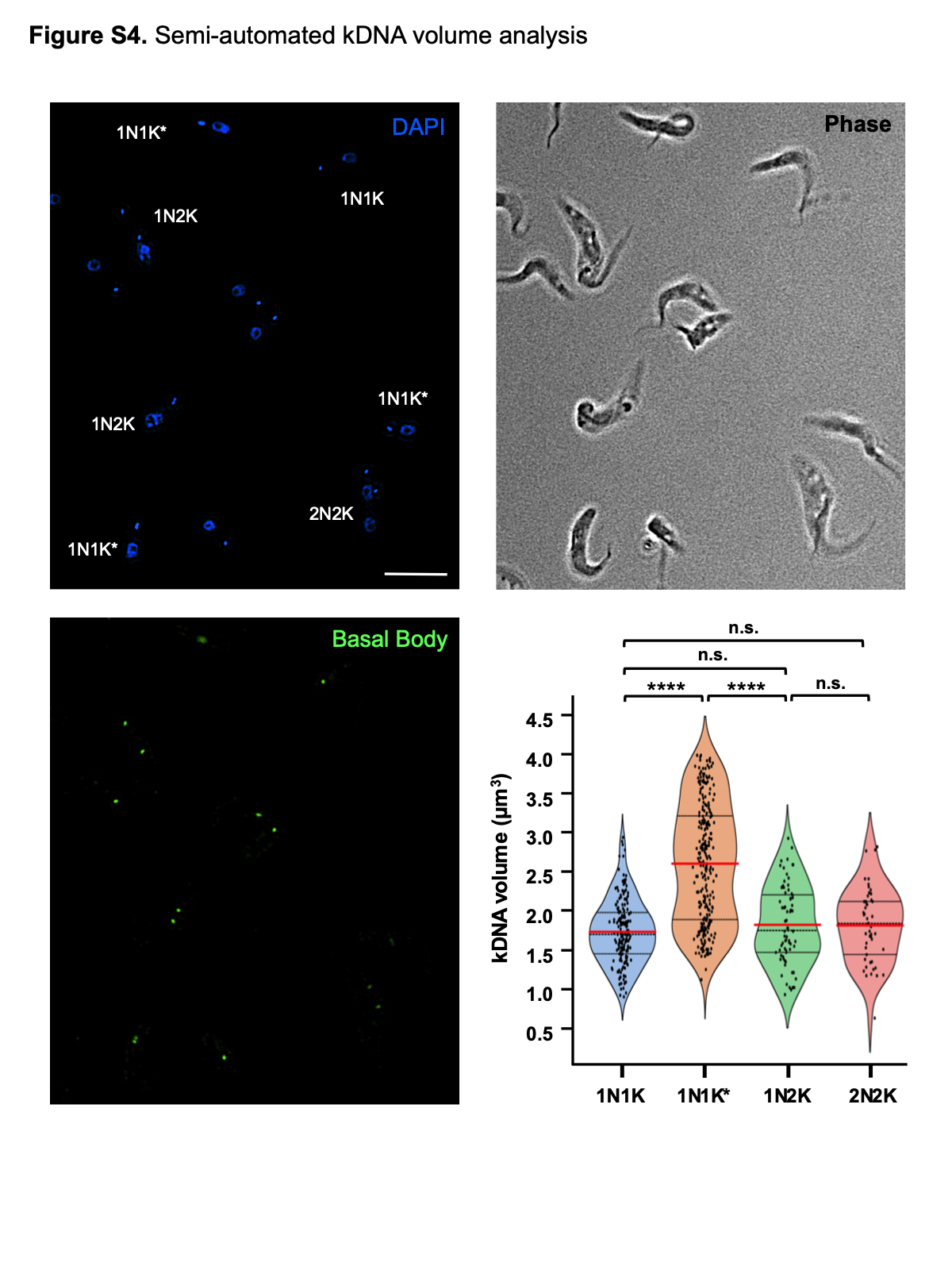

Supplement: S4 Fig — Representative images of asynchronous SMUMA cells. DNA was stained with DAPI (blue) and basal bodies were detected using YL1/2 (green). Multiple karyotypes are indicated in DAPI image. Scale bar, 20 μm. Quantification of kDNA volume from 580 DAPI stained cells. The red line indicates the mean of the kDNA volume for each karyotype. ****: P value <0.0001, as calculated by an unpaired two-tailed t test. (TIF) [file pone.0321334.s006.tif]

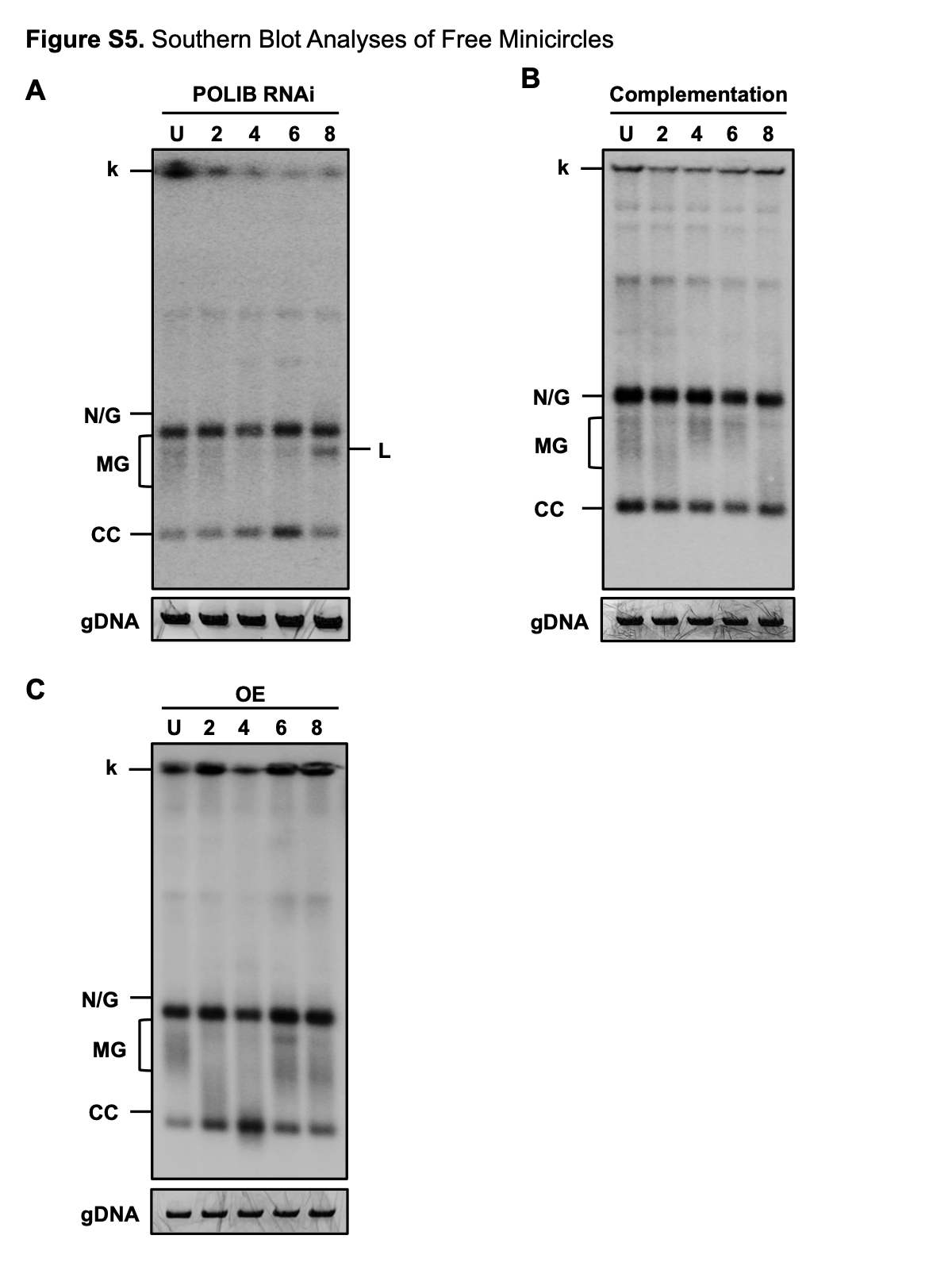

Supplement: S5 Fig — (A) Representative Southern blot showing the changes in free minicircles at selected induction points for IBCompVaT (clone P2D11) grown in the absence or presence of 250 µM Van. (B) Same as (A) but for IBCompVaT (clone P2D11) grown in the absence or presence of 250 µM Van and 4 μg/ml Tet. (C) Same as (A) but for IBCompVaT (clone P5D1) grown in the absence or presence of 4 μg/ml Tet. Abbreviations for all blots: k, kDNA network; N/G, nicked/gapped; MG, multiply gapped; CC, covalently closed; L, linearized; gDNA, loading control. (TIF) [file pone.0321334.s007.tif]
