# Supplementary material for: An independently tunable dual control system for RNAi complementation in Trypanosoma brucei
Source: PLoS One. 2025 May 12;20(5):e0321334. doi: 10.1371/journal.pone.0321334 (PMC12068568; doi:10.1371/journal.pone.0321334)

Raw Images

The red box highlights the cropped representative image selected for the figure, while the black box highlights the area of interest used for quantification.

Data not used in this study or unrelated to the figure are labeled **X**.

Figure 1B (western blots)

+Tet (days) for all blots

Replicate 1

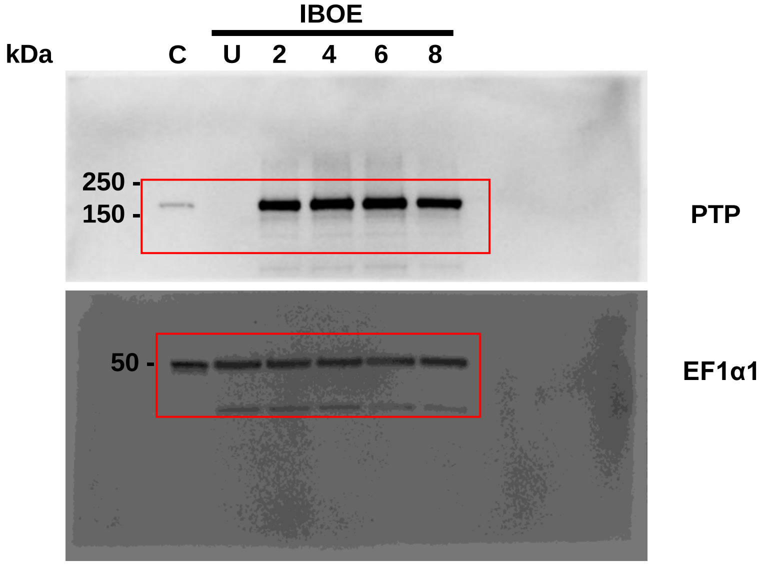

Replicate 2

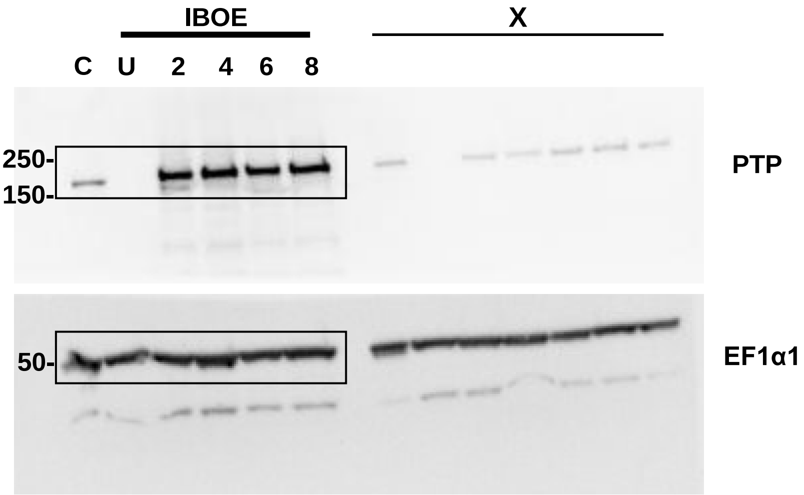

Replicate 3

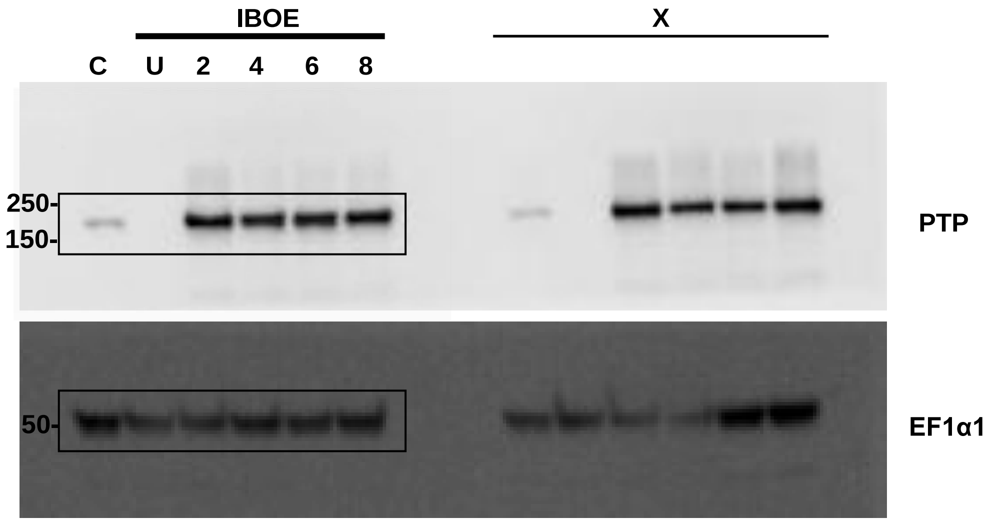

Figure 1E (western blots)

+Tet (days) for all blots

Replicate 1

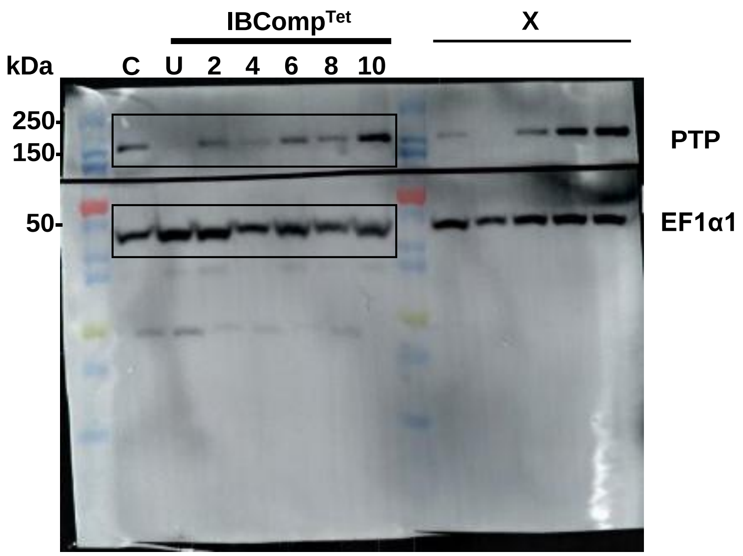

Replicate 2

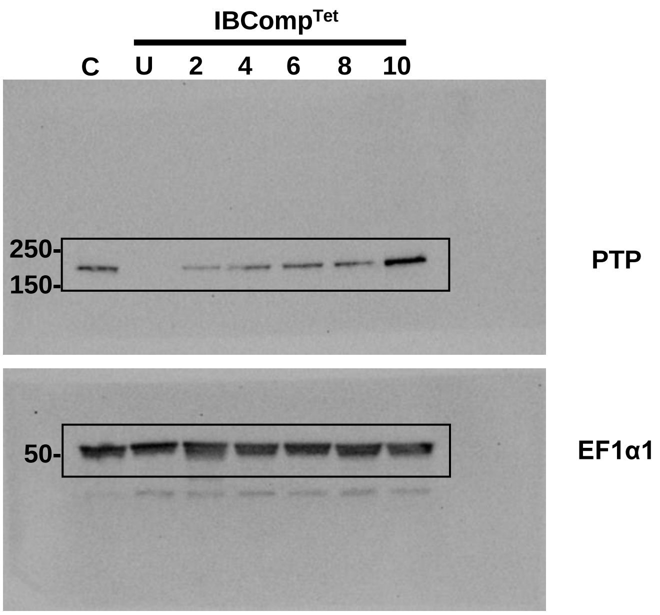

Replicate 3

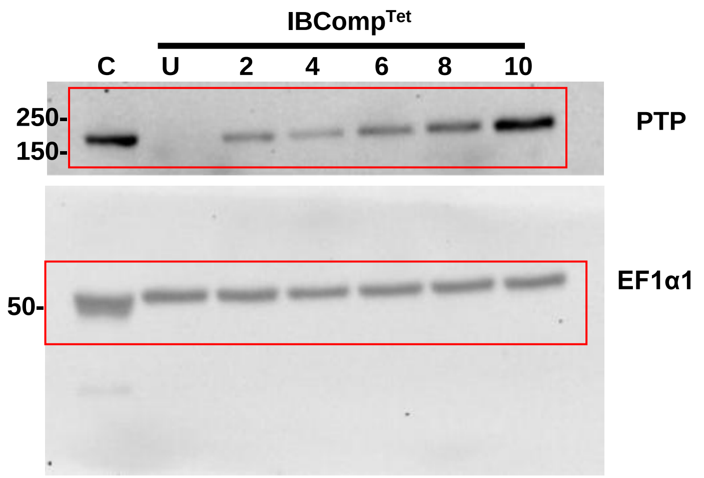

Figure 2B (northern blots)

+Van or +Tet (days) for all blots

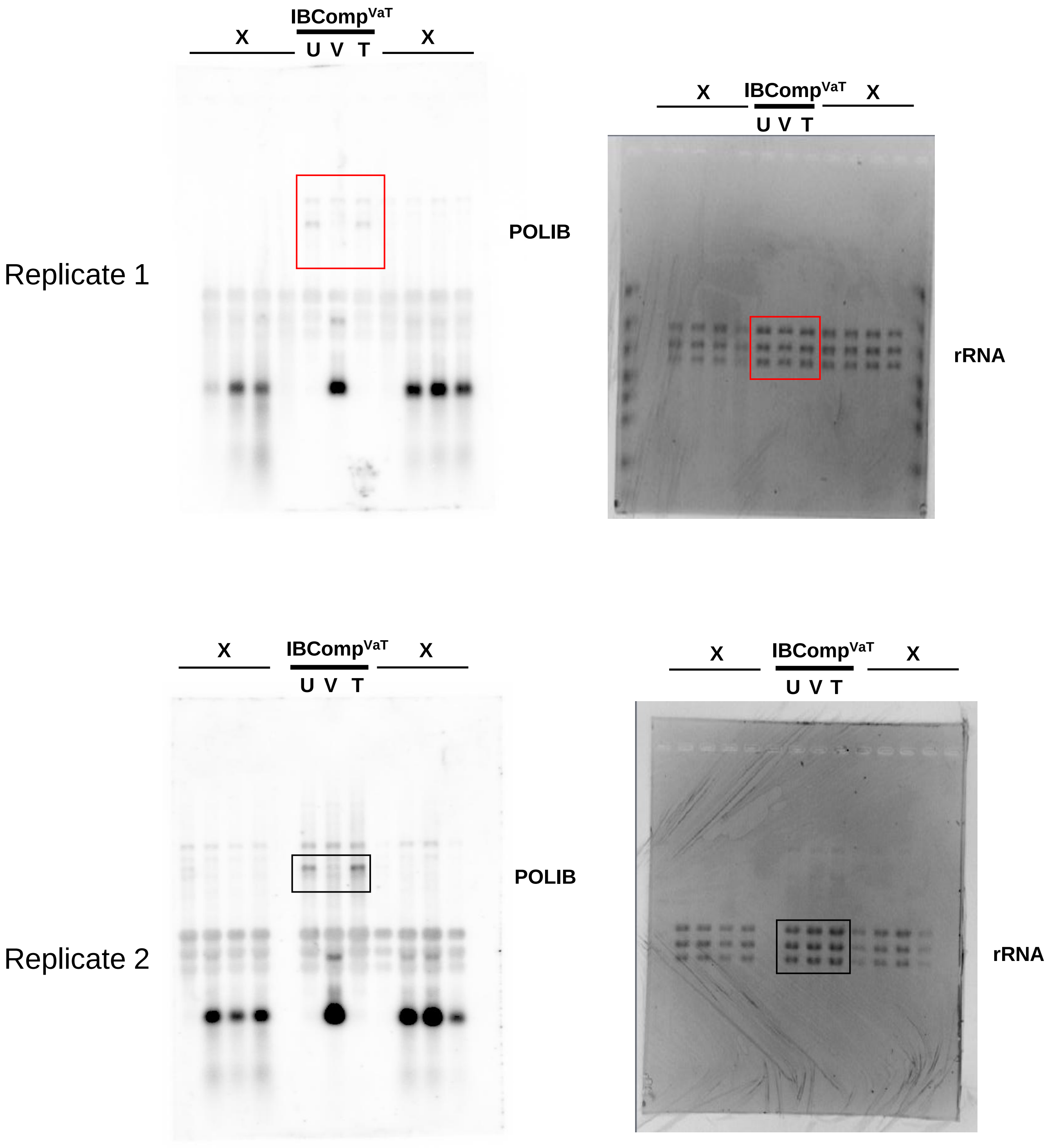

Figure 2C (western blots)

+Van (days) for all blots

Replicate 1

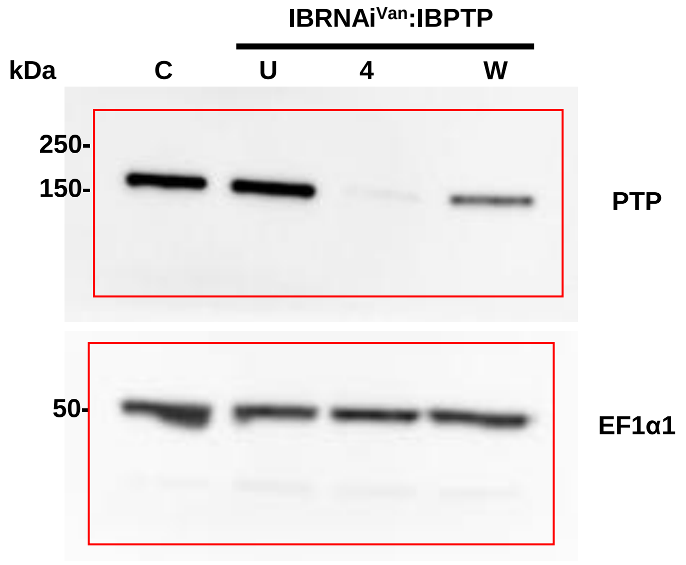

Replicate 2

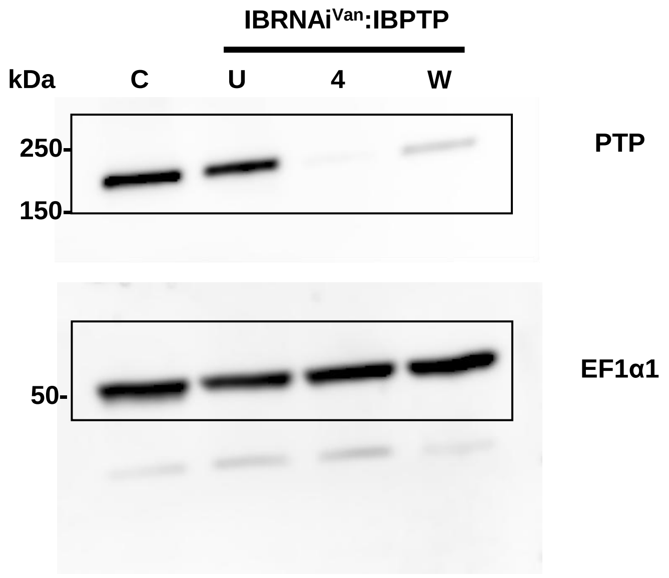

Figure 2D (western blots)

+Van or +Tet (days) for all blots

Replicates 1 and 2

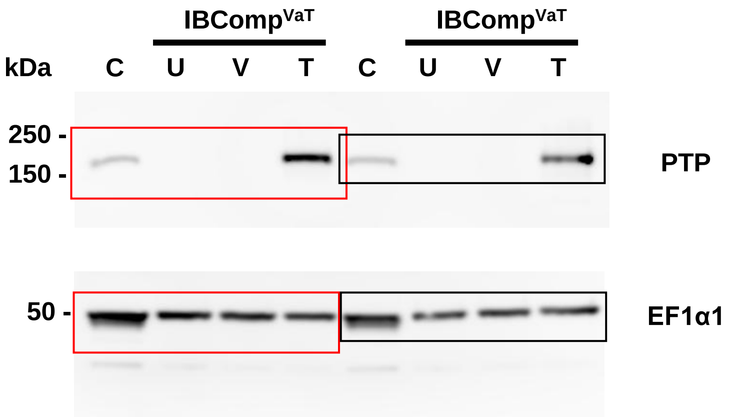

Membrane was cut to develop separately.

Figure 4B (western blots)

+Tet (days) for all blots

Replicate 1

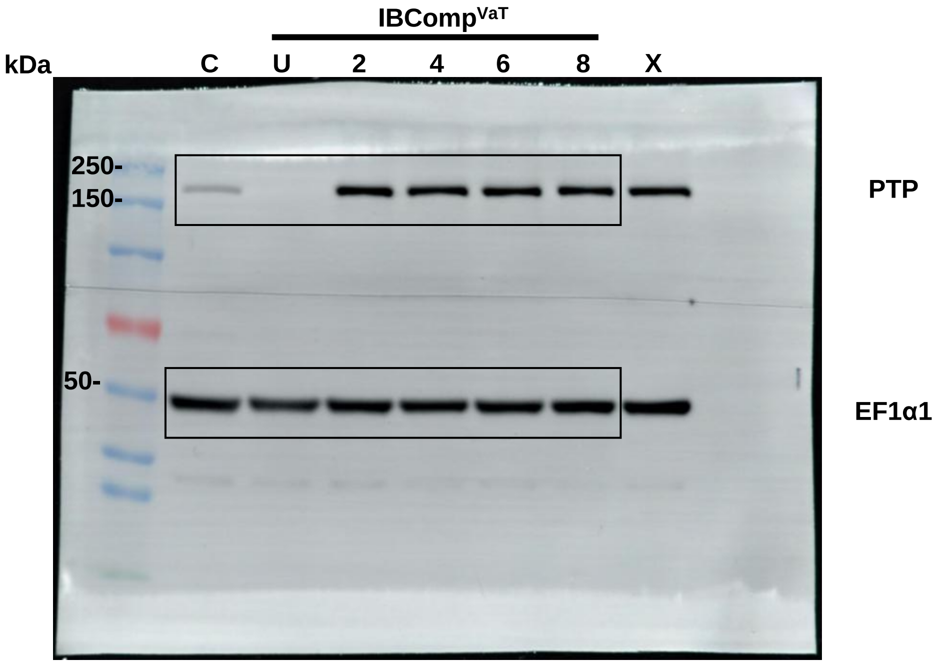

Replicate 2

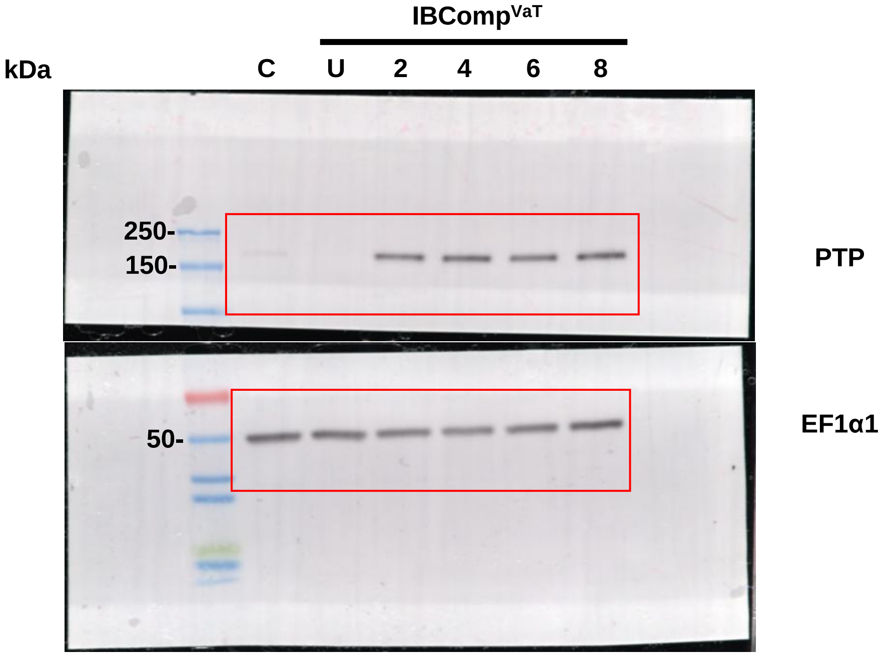

Figure 4D (northern blots)

+Van and/or +Tet (days) for all blots

Replicates 1 and 2

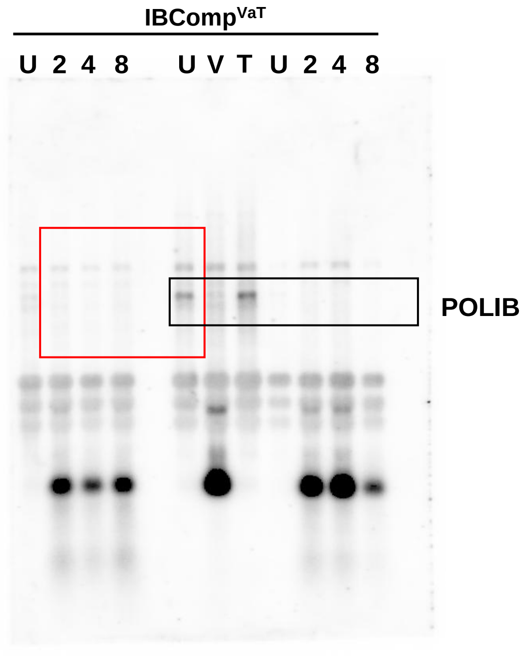

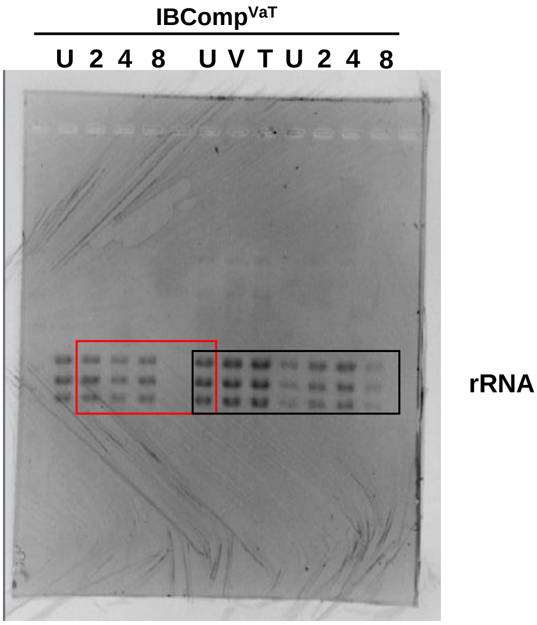

Figure 4E (western blots)

+Van and +Tet (days) for all blots

Replicate 1

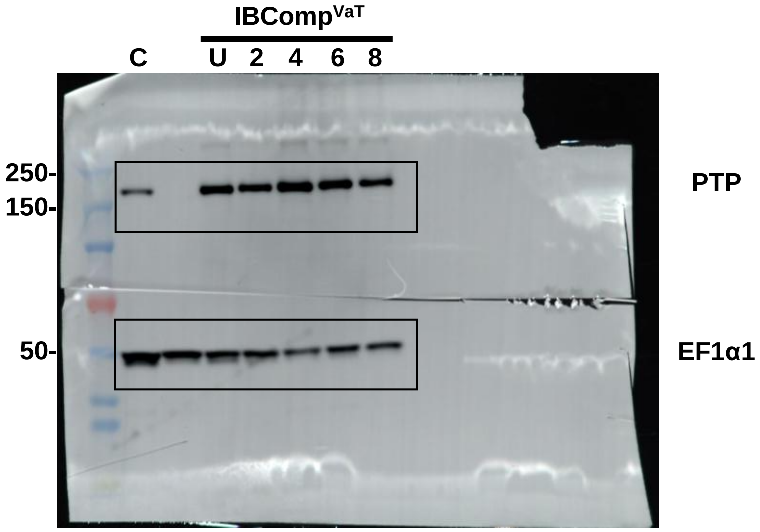

Replicate 2

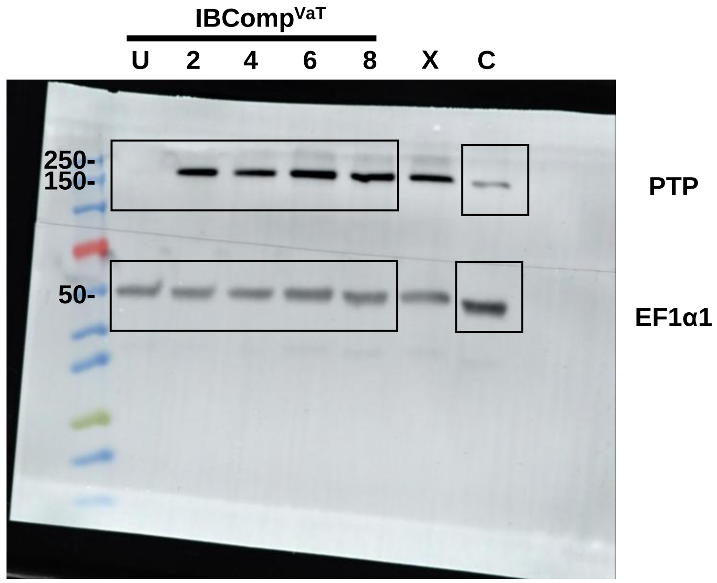

Replicate 3

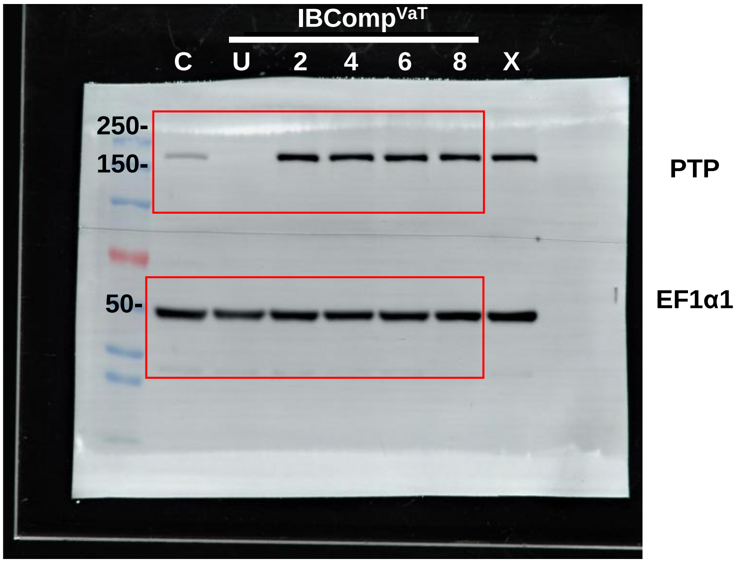

Figure S1B (western blots)

+Tet (days) for all blots

Replicate 1

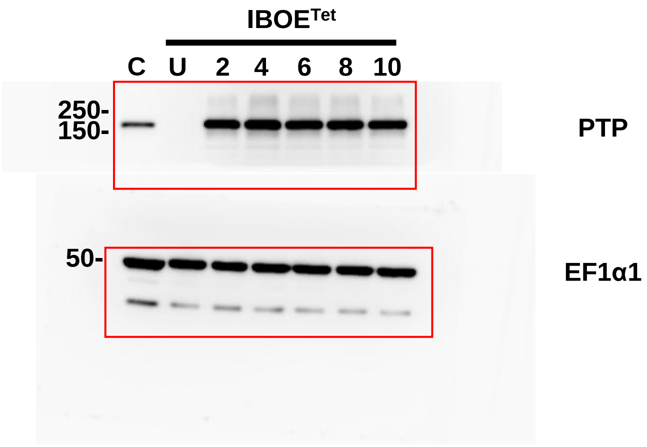

Replicate 2

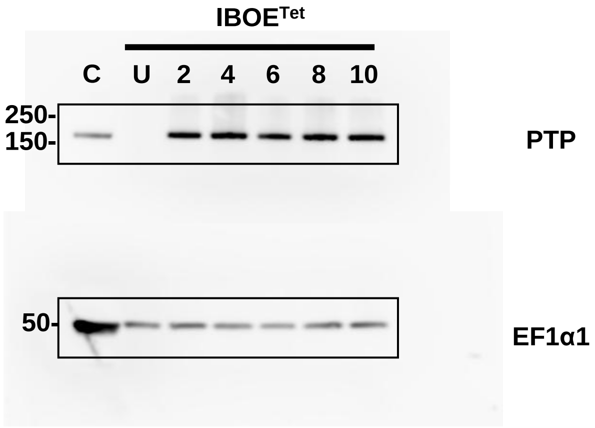

Figure S1D (western blots)

+Tet (days) for all blots

Replicate 1
1 µg/ml

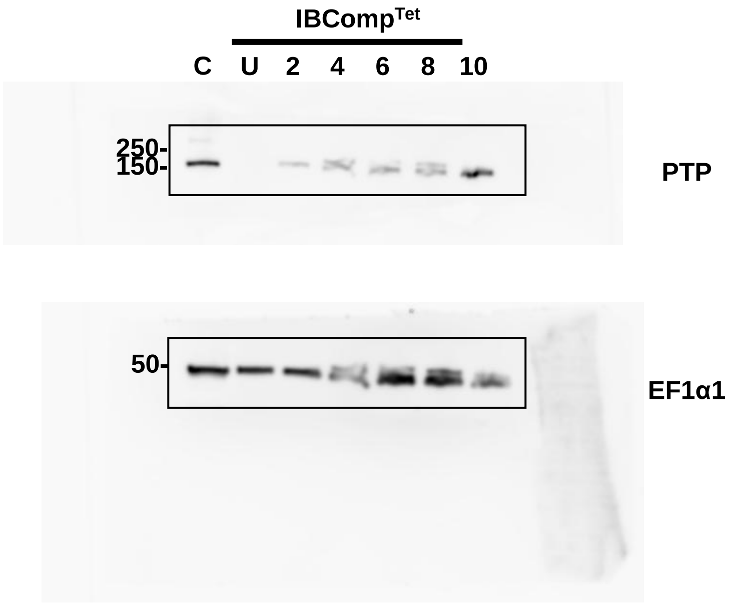

Replicate 2
1 µg/ml

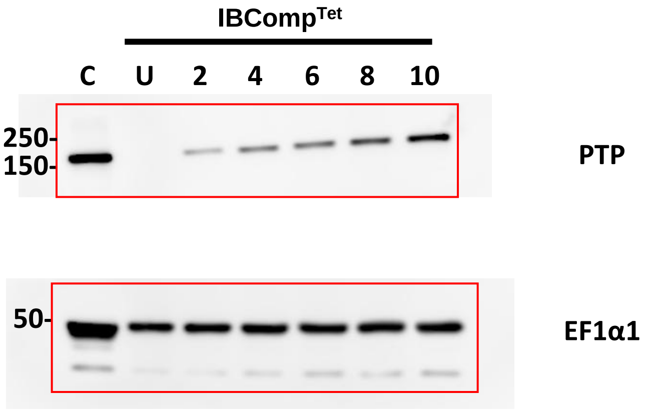

Figure S1D continuation (western blots)

+Tet (days) for all blots

Replicate 1
2 µg/ml

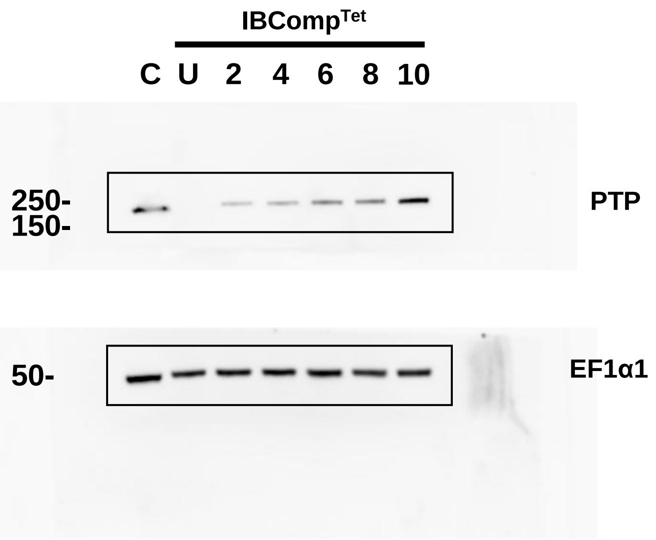

Replicate 2
2 µg/ml

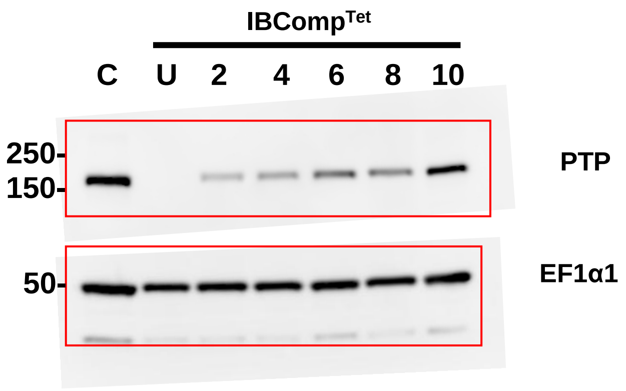

Figure S1E (northern blot)

+Tet (days) for all blots

Replicates
1 and 2

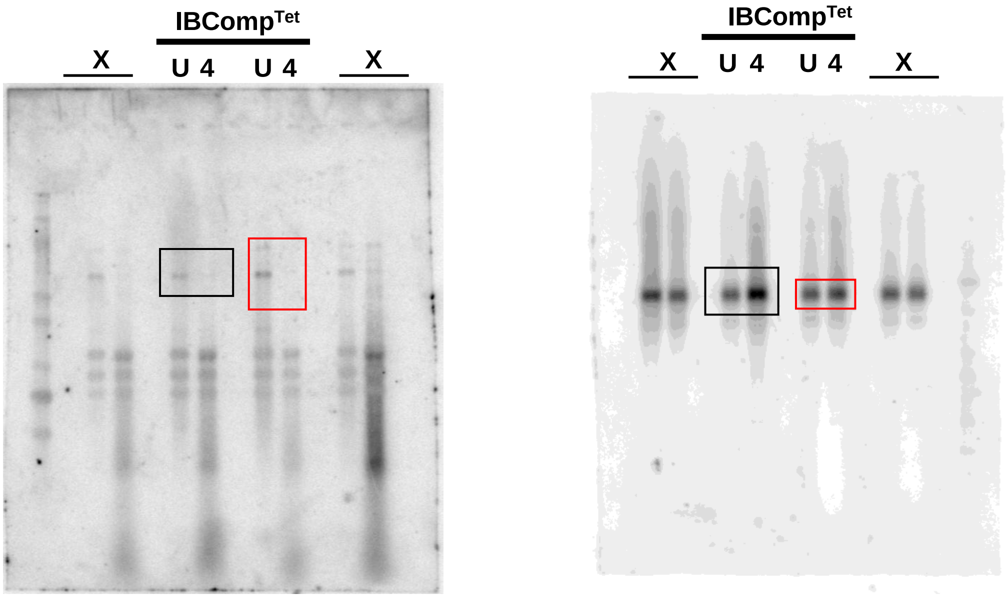

POLIB specific probe

Tubulin specific probe

Figure S2A (western blot)

Replicate 1

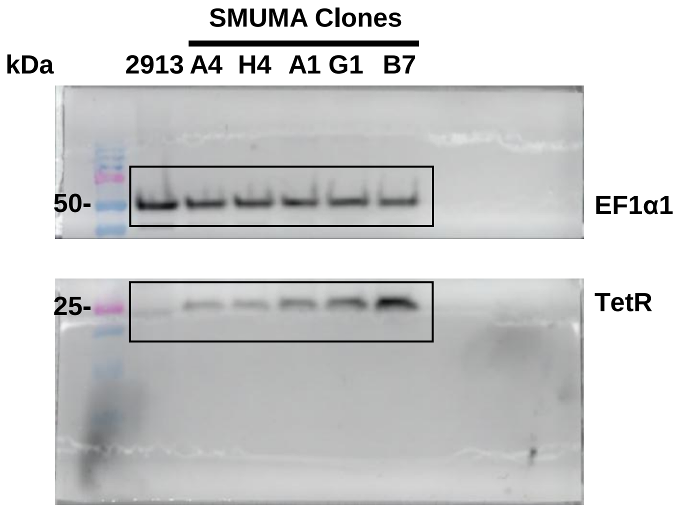

Replicate 2

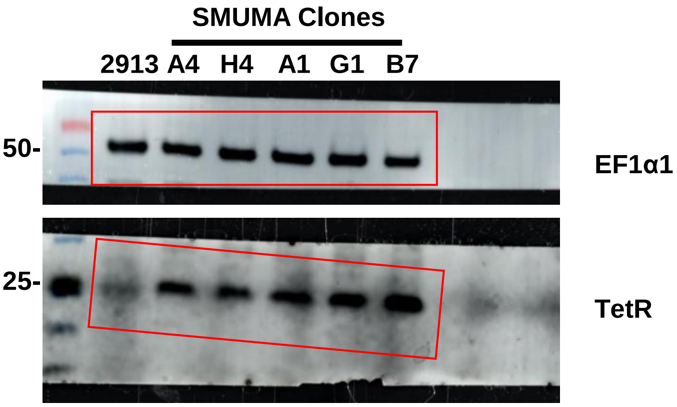

Figure S2B (northern blot)

Replicate 1, 2 and 3

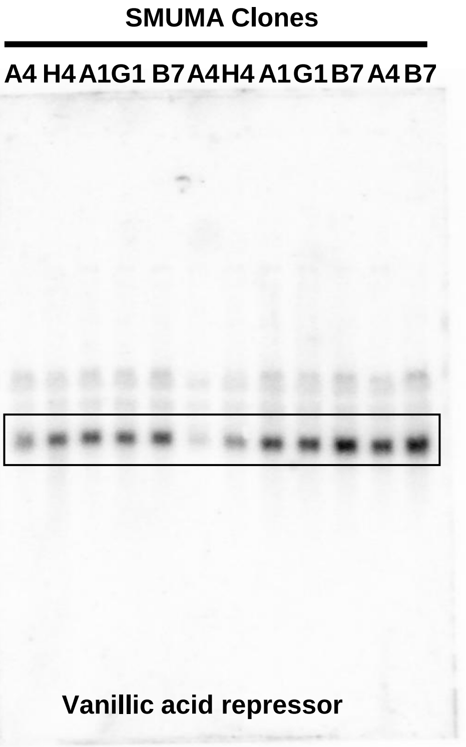

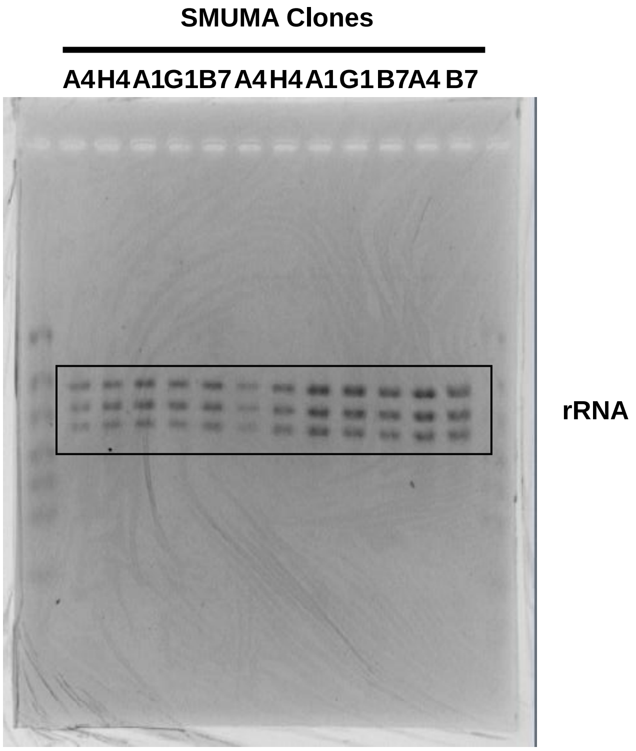

Replicate 4

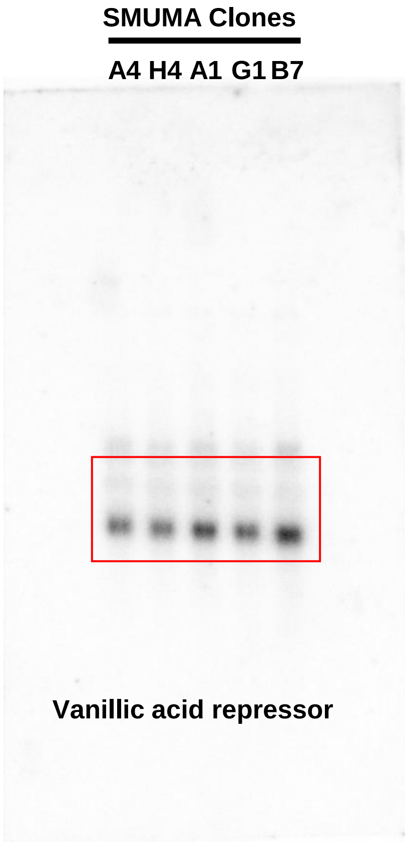

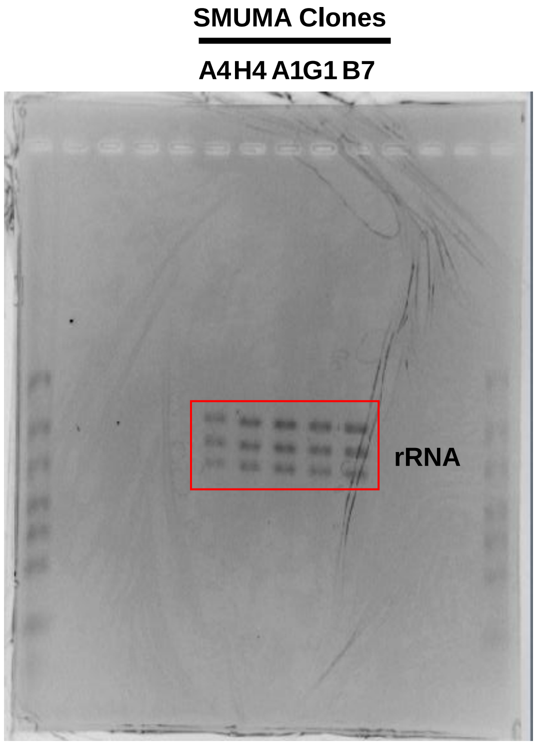

Figure S3A (northern blot)

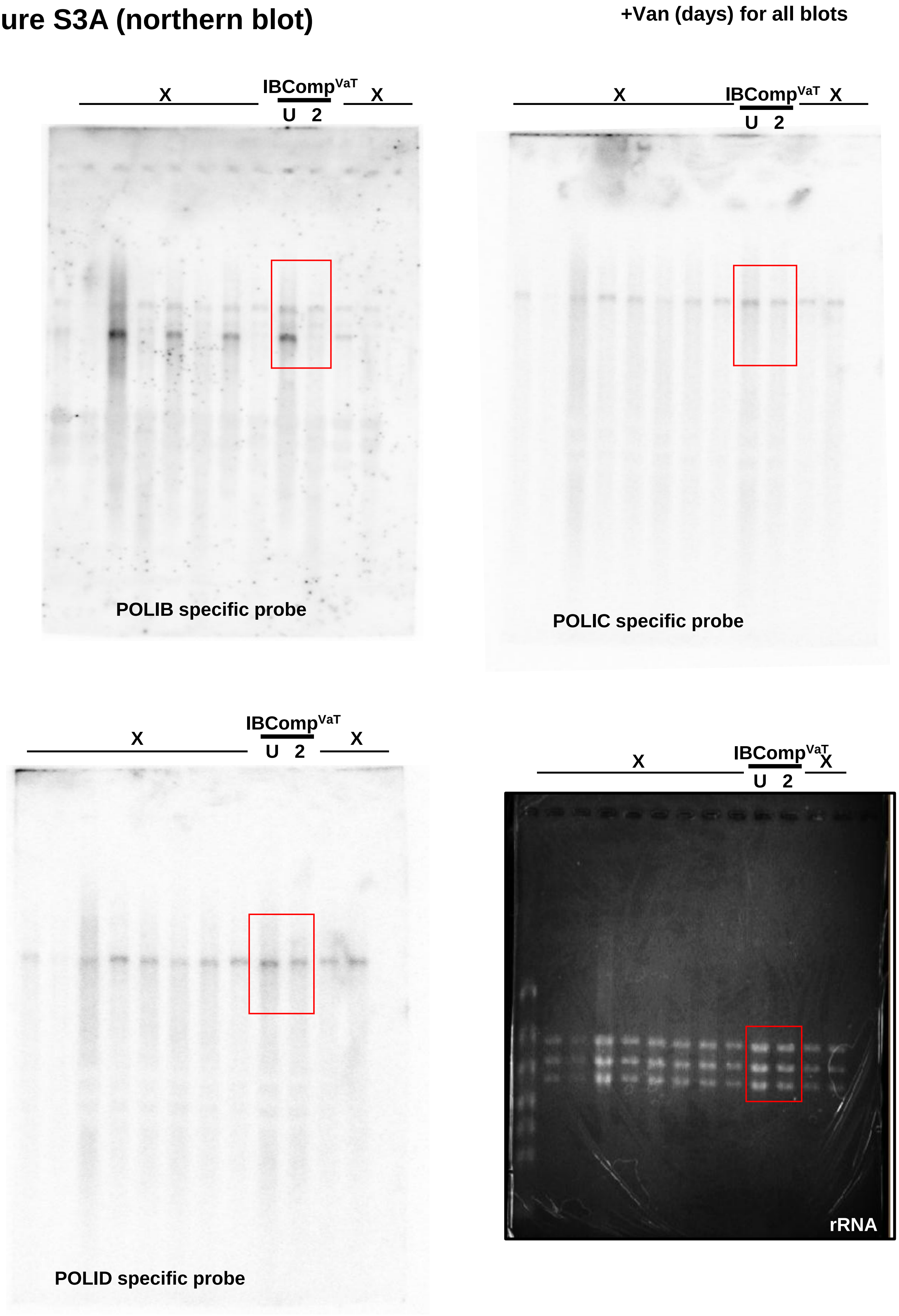

Figure S3B (western blot)

+Van (days) for all blots

Replicates
1 and 2

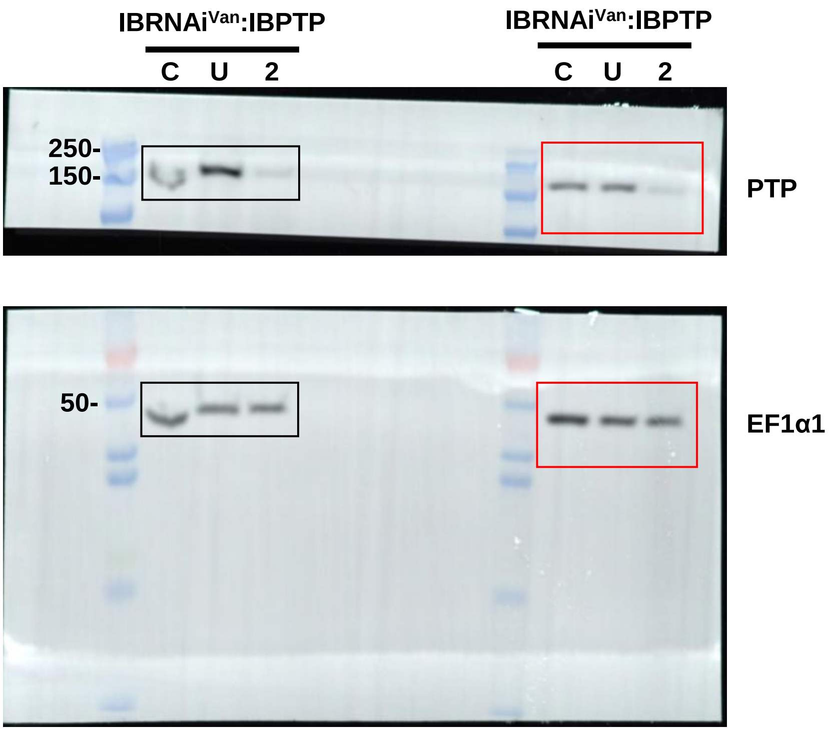

Figure S5 (RNAi Southern blot)

+Van(days) for all blots

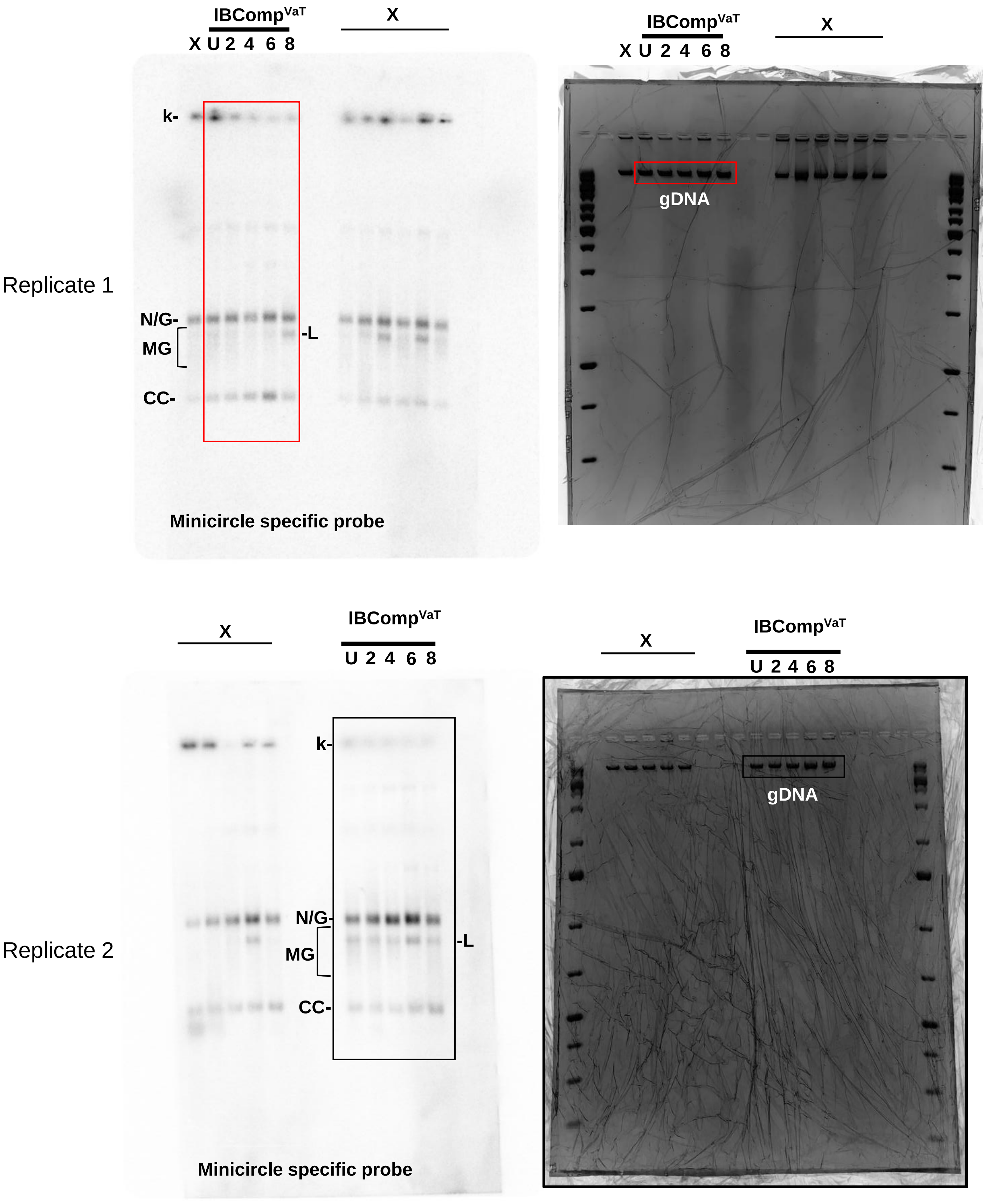

Figure S5 continuation (RNAi Southern blot)

Replicate 3

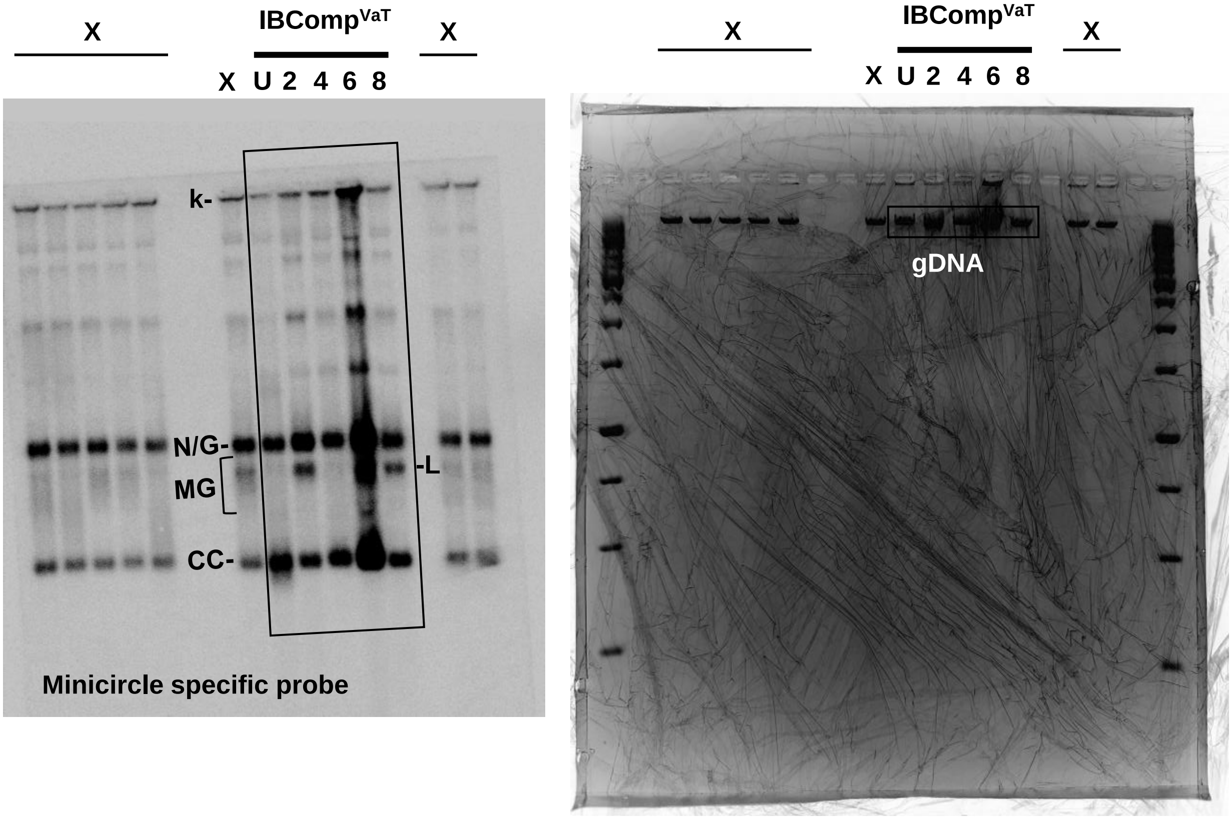

Figure S5 continuation (Complementation Southern blot)

+ Tet and +Van(days) for all blots

Replicate 1

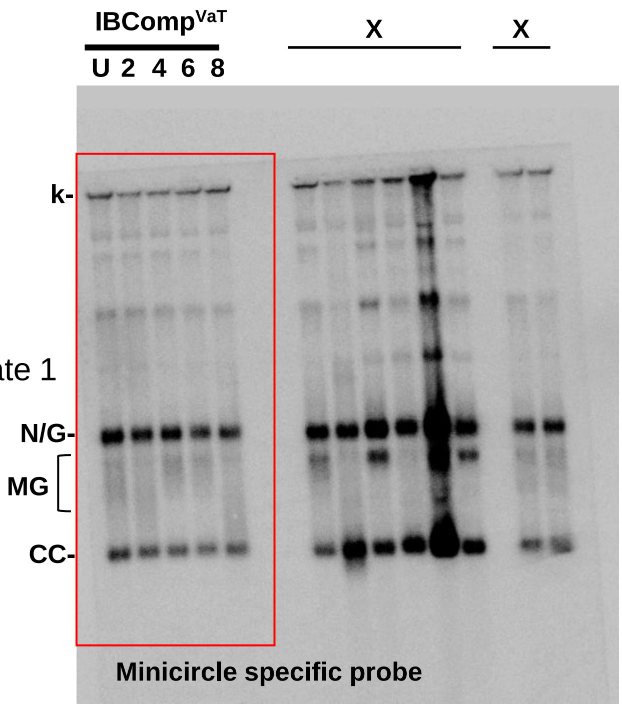

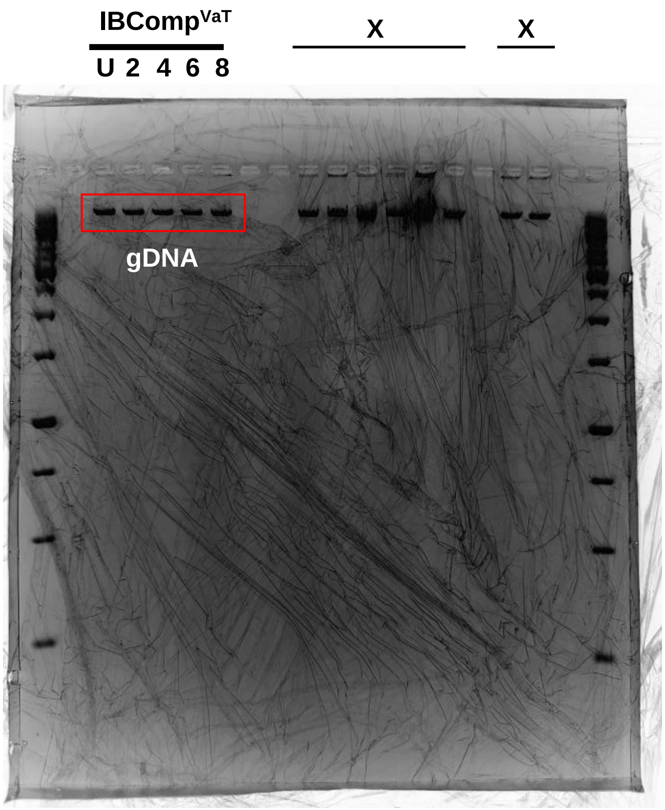

Replicates 2 and 3

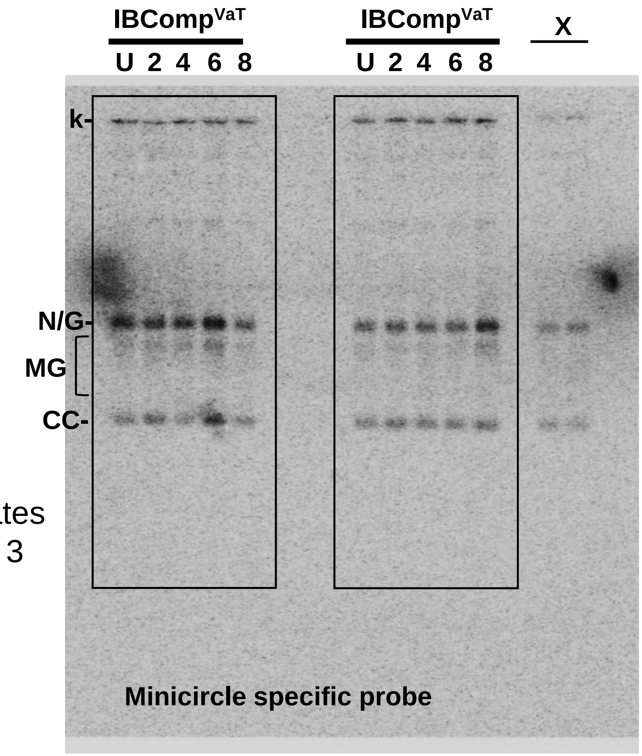

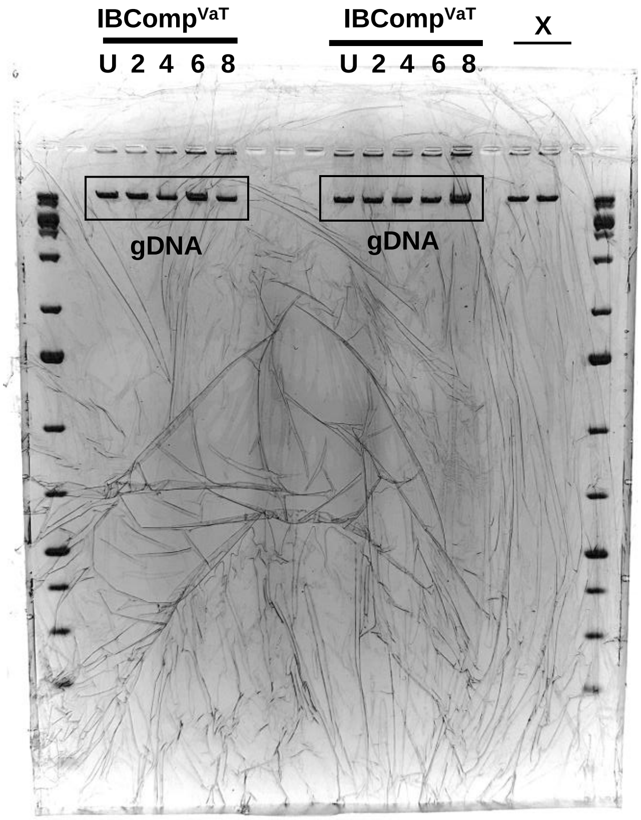

Figure S5 continuation (Overexpression Southern blot)

+Tet (days) for all blots

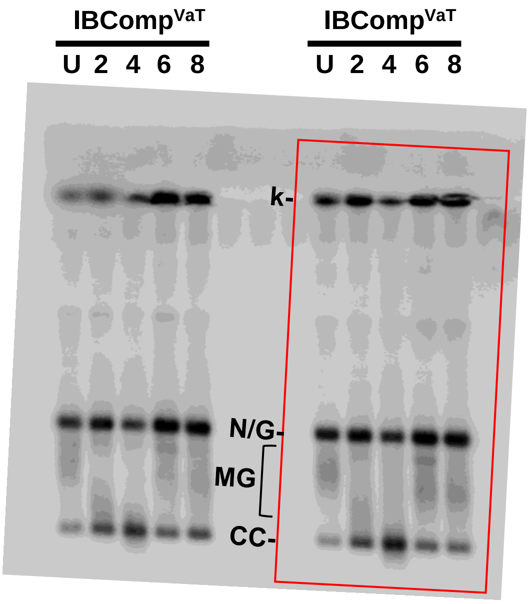

Replicates 1 and 2

Minicircle specific probe

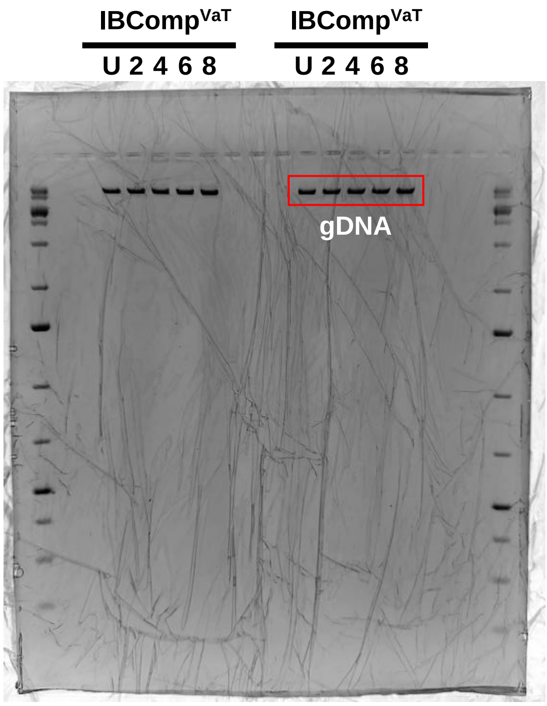

Supplement: S1 Raw Images — (PDF) [file pone.0321334.s008.pdf]
